# Conditional intensity/point process model of task-related prefrontal spiking: effects of performance

**DOI:** 10.1186/1471-2202-12-S1-P160

**Published:** 2011-07-18

**Authors:** David M Devilbiss, Craig W Berridge, Rick L Jenison

**Affiliations:** 1 Psychology Department, University of Wisconsin, Madison, WI 53705, USA

## 

**Figure 1:**
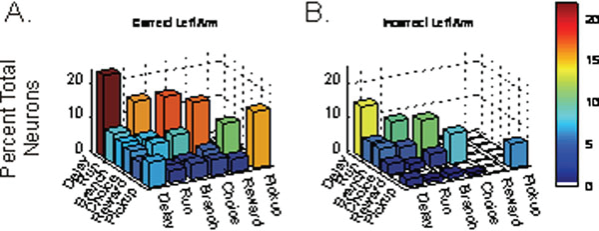
Heterogeneous plPFC neurons participate in and multiplex multiple functional ensembles. Plotted are the percentages of neurons for which task event-states (see Supplemental Material, Fig. S4) associated with this task significantly predicts the spiking activity of these cells. Colors and bar height directly reflect the percent of neurons representing each event-state. A.) Event-states related to correct trials with a left spatial goal. B.) Plots of event-states during error trials. The diagonal of each panel represents the number of neurons significantly related to a single event-state. Off-axis bars are the 1^st^ order interactions such that values represent the number of neurons shared between these cell assemblies. Delay intervals are represented by the largest neural assemblies. Error trials exhibit deteriorated and smaller cell assemblies.

The spiking activity of neurons in the prefrontal associational cortex (PFC) of animals engaged in cognitive tasks is posited to reflect diverse set of independent cognitive and behavioral processes required for the successful attainment of future goals. Additionally, a neural representation of past information (i.e. spike history) is likely critical for performance in these delayed-response tasks of working memory. In the current study, we used a conditional intensity - generalized linear model (CI-GLM) statistical framework to determine the degree to which spike history and components of a delayed-response task predict PFC neural spiking as opposed to traditional peri-event time histogram (PETH) analysis approaches. The final CI-GLM model takes the form,, such that the conditional intensity function *λ*(*t*|*H_t_*) is predicted by a generalized linear model from a series covariates where (*µ*) is the intercept of the equation representing the background level of activity,  represents an autoregressive process of order (*K*), and  is the design matrix of each event-state (*r*) for each sample in time (*t*) of the behavioral task and *β_r_* are the estimated weighting parameters. The discretized time for increasing spiking history durations is represented as Δ*N_t–k_* with *γ_k_* representing the autoregressive coefficients. We found that in rats performing a PFC-dependent delayed-alternation task of working memory, the vast majority of PFC neurons participate in the representation of more than one task-related event-state (multiplexer neurons). Additionally, the size of functional neural groups or cell assemblies was critically linked to the level of behavioral performance (Fig. 1). Poor behavioral performance was not solely associated with an attenuation of spiking activity, but instead the activation of inappropriate assemblies during error trials may actively direct the animal towards an incorrect choice. Furthermore, we demonstrate that spike history improves predictions of neural activity within the PFC. In particular, the event-state most strongly associated with spike history effects was when animals revealed the decision to enter a particular maze arm. Combined, these and other results suggest that PFC spiking activity may reflect an overarching action-plan or policy rather than multiple independent cognitive processes.

